# Design and analysis of UPQC in a microgrid using model reference adaptive control ensemble with back-stepping controller

**DOI:** 10.1016/j.heliyon.2024.e34140

**Published:** 2024-07-09

**Authors:** Sandip Kumar Das, Sarat Chandra Swain, Ritesh Dash, Jyotheeswara Reddy K, Dhanamjayalu C, Ravikumar Chinthaginjala, Ramakanta Jena, Hossam Kotb, Ali ELrashidi

**Affiliations:** a School of Electrical Engineering, KIIT Deemed to be University, India; b School of Electrical and Electronics Engineering, REVA University, Bengaluru, India; c School of Electrical Engineering, Vellore Institute of Technology, Vellore, India; d School of Electronics Engineering, Vellore Institute of Technology, Vellore, India; e Department of Electrical Engineering, Seemanta Engineering College, Mayurbhanj, Odisha, India; f Department of Electrical Power and Machines, Faculty of Engineering, Alexandria University, Alexandria 21544, Egypt; g Electrical Engineering Department, University of Business and Technology, Ar Rawdah, Jeddah, 23435, Saudi Arabia

**Keywords:** Backstepping control, ANFIS, Fuzzy, DC-microgrid, Point of interconnection

## Abstract

In recent years, the power sector has shifted to decentralized power generation, exemplified by microgrids that combine renewable and traditional power sources. With the introduction of renewable energy resources and distributed generators, novel strategies are required to improve reliability and quality of power (PQ). In our proposed system, a model consisting of photovoltaics, wind energy, and fuel cells has been designed to share a network, bolstered by the integration of UPQC to rectify PQ issues. Notably, our model introduces a Back-stepping controller method featuring Model Reference Adaptive Control (MRAC) with online parameter tuning, offering superior adaptability and responsiveness. This approach not only ensures optimal grid management but also enhances efficiency and stability. Furthermore, the proposed model demands minimal additional infrastructure, leveraging existing resources to streamline implementation and maintenance, thereby promoting sustainability and cost-effectiveness. The research culminates in a comparative analysis between the MRAC-Back-stepping controller, Adaptive Neuro-Fuzzy Inference System (ANFIS), and Fuzzy controller, highlighting the efficacy and versatility of our proposed model in microgrid operations. A Matlab model has been designed along with a hardware setup to demonstrate the robustness of the model.

## 1 Introduction

Recently, the modern world has paid more and more attention to population growth and environmental changes. Population growth and industrialization require more energy from the grid [1], [2]. Traditional energy is not enough to meet the demand, and it also increases the impact on the environment. The rapidly increasing energy demand and environmental issues have attracted people's attention. The microgrid is a new way of generating energy without pollution at the distribution level [3], [4]. Microgrid is nothing but integrating multiple distributed energy sources, storage systems, and various power converters to control the flow of energy between the grid and consumers [5], [6].

Power electronic converters play an important role in controlling the microgrid and connecting the network to the microgrid [7]. They not only improve power quality because fast switching can enhance harmonics, but also provide reactive power support in the network to which the microgrid is connected [8]. The rapid development of power electronics technology and its applications has greatly changed the characteristics of power distribution systems. Devices/loads based on power electronic devices act as non-linear components and cause serious PQ problems in today's power distribution systems [9].

Distributed Energy Resources (DERs) are nothing more than small energy sources that can be used in groups to provide enough energy to balance supply and demand [10]. Because renewable energy has excellent economic, environmental and technological advantages, it has attracted the attention of many researchers [11]. Due to these advantages, the renewable-based generation system's penetration into the existing traditional network is rapidly increasing [12]. Generally, DERs have more connections with the distribution grid than with the transmission grid. There are many types of RES, such as wind, solar photovoltaic, micro-turbines, fuel cells, etc., and they have different sizes. In addition, due to the deregulation of the energy market, the penetration of DER in many countries has increased significantly. However, as their numbers continue to grow, better energy management methods are also needed [13].

Management practices play an important role in the success of microgrids. Depending on the operating mode of the microgrid, several control elements must be addressed [14]. In grid-connected mode, it is crucial to independently control active and reactive power. In this mode, the control of voltage and frequency becomes a priority [15]. Only when these control measures are implemented correctly the microgrid be utilized as a reliable power source. Proper control ensures the stability of the system. In this work, reference signals will be generated and the controls will be adjusted to follow them to achieve the desired performance. The author suggests that a suitable control system with multi-agent control will help optimize energy management in connected and isolated modes [16]. Although it has been proven that real-time control can be achieved, the control strategy is very complex. Microgrid provides a powerful solution that combines the most advanced components and provides technology to meet load requirements. A decentralized management architecture based on agency decisions is proposed. Protecting microgrids is another topic worthy of attention. Protection should be focused on self-sufficiency and networked operations. In some cases, admittance relays are used, which have inverse time-based characteristics on the line admittance measured [17].

Innovations in power electronics technology have increased the awareness of power quality (PQ) in power distribution systems. According to the standard, the term PQ is defined as the physical characteristics of a power source that supplies power under normal operating conditions that do not affect end-user performance. By connecting the multifunctional inverter to the grid, the PQ problem of the microgrid can be enhanced. Harmonic distortion, maintaining active and reactive power, and voltage fluctuations are the main problems of PQ in microgrids. Generally, power quality problems have a wide range of classifications, which are related to voltage and current. Any deviation from the rated voltage or any distortion on the power supply side is called voltage-dependent PQ distortion. Overloads, power failures, opening/closing large loads and capacitor banks, and distribution transformer overload all cause voltage-related PQ distortion, voltage unbalance, harmonic distortion, etc [18]. Power electronic equipment behaves like a non-linear load and will produce unwanted PQ distortion, but interestingly, power electronic equipment itself provides a solution for PQ distortion. In the current situation, compensation equipment must be designed because it also plays a dual role in protecting sensitive loads from PQ voltage-related distortion [19].

Improving network quality in microgrid systems with nonlinear loads is a difficult task today. The main focus of the proposed research is the design of UPQC with the best compensation capability to protect the microgrid and sensitive loads from various PQ distortions at the same time. The best compensation capability of UPQC is achieved through control strategies. The traditional control method, that is, a PI controller with a fixed gain, is used to adjust the intermediate circuit voltage to a constant reference value. In a distorted environment, a fixed-gain PI controller will have poor DC bus control voltage [20]. Based on the previous discussion, a lot of work has been done to develop power management and control systems for microgrids, but there is still a lot of room for development to achieve better performance and higher reliability. Based on the information in the bibliographic overview, UPQC systematically classifies power supplies and converters. This general study shows that different UPQC configurations need to be used to compensate for power quality issues in three-phase three-wire systems and three-phase four-wire systems [21].

The overview shows that there is not much work found in the literature in using UPQC to improve the network quality in autonomous microgrid systems. Therefore, this paper focuses on improving the PQ of the microgrid system through various control methods and evaluates the performance of the UPQC that connects various unconventional power supplies through the UPQC intermediate circuit capacitor. The literature review indicates that UPQC works better if the intermediate circuit voltage is kept constant. Through the intermediate circuit side connection of UPQC, the effect of improving PQ in suppressing harmonics was observed [22].

An integral backstepping controller along with an energy storage system has been investigated by using PV, Wind and HESS. The main aim of the researcher is to coordinate the DC voltage and thereby control the DC microgrid. The author investigated the energy management algorithm covering high-level control such as shortage mode and excess mode to ensure the power balance [23].

The authors in their research proposed a non-linear lyapunov-based control approach by using a backstepping controller to estimate the unknown parameters and to regulate the dc-voltage of the dc-bus under changing environment conditions. The method also ensures the adaptation of state variables at each operating point. The validity of the model was tested on a dc-microgrid [24], [25].

A dc-centralized non-linear backstepping controller has been investigated by authors. They have used Lyapunov theory to understand the dynamic behavior of inverter based distribution generation. The authors have validated the proposed controller in terms of MATLAB simulation and hardware-in-loop experiment. However, the research does not explain the hybrid DC-AC converter.

In another research, the authors used an observer based backstepping sliding mode controller to regulate the DC-DC converter. The proposed model provides less steady state error with a fast transient response. It is also noticed from their research that, the integrated area error is also very less as compared to the other Controller based microgrid approach [26]. The comparative analysis of different literature review is given in Table 1.

**Table 1:** Comparative analysis of different literature review.

Paper	Contribution	Results	Limitations
[27]	Examination of UPQC effectiveness in mitigatingpower quality issues	The effectiveness of UPQC is examined for a variety of PQ	Power electronic devices degrade power quality.
[28]	UPQC reduces power quality issues like harmonics and sag.	Study of combination of series and shunt active filters	Growing number of applications make methods ineffective
[29]	Implemented fuzzy logic-based controller for DC link control	Hybrid UPQC with distributed generation reduces power quality problems	The current state of power quality issues in industrial environments is not mentioned in the provided paper.
[30], [31]	Use of a weighted feedback algorithm to manage PCC parameters and UPQC performance.	The paper introduces a unified power quality conditioner based on the VSC theorem.	The model is restricted to static analysis

Based on the above literature review, the following objectives have been identified as research goals for UPQC implementation in a microgrid.

• Development of model reference adaptive control technique for the backstepping controller to integrate the UPQC in a microgrid.
• Controlling the tuning parameter by using online adoption.
• To compare the performance of the proposed controller with the classical PI controller

This research introduces innovative control methodologies utilizing a Back-stepping controller combined with Model Reference Adaptive Control (MRAC) to enhance power quality (PQ) in microgrid systems integrating renewable energy sources. The study highlights the design of a Unified Power Quality Conditioner (UPQC) adept at addressing PQ issues like voltage fluctuations and harmonic distortions, which are particularly prevalent with high penetration of distributed energy resources (DERs) like photovoltaics, wind energy, and fuel cells. Notably, the paper proposes a novel control strategy that allows for online parameter tuning, offering a dynamic response to fluctuating grid conditions and improving the adaptability and efficiency of microgrid operations. This approach contrasts with traditional static control systems, promoting enhanced system stability and operational reliability with minimal infrastructure modifications.

The remainder of the paper is organized as follows. Section 2 presents the problem formulation and mathematical modeling, while Section 3 introduces the controller methodology. Section 4 provides a detailed description of the experimental setup, followed by Section 5, which presents the analysis and discussion of the results. Finally, the conclusion is presented in Section 6.

## 2 Problem formulation

The microgrid has gained the attention of power engineers as a means to enhance the stability, reliability, and efficiency in power distribution systems. Again, interconnection of these resources into the grid introduces other challenges in power quality, such as fluctuation of voltage and injection of harmonics at the point of common coupling. To address such a problem, in this research work, UPQC has been proposed as a solution. In order to increase the voltage stability and reduce inter-harmonic oscillations, an attempt has been made to develop a model reference adaptive control (MRAC) ensemble with a backstepping controller for UPQC. The proposed control logic ensures a smooth transition of energy flow between the distributed energy resources. Therefore, in the first place, a detailed mathematical model of a microgrid consisting of all distributed energy resources such as load and generation has been developed [32].

**Figure 1:**
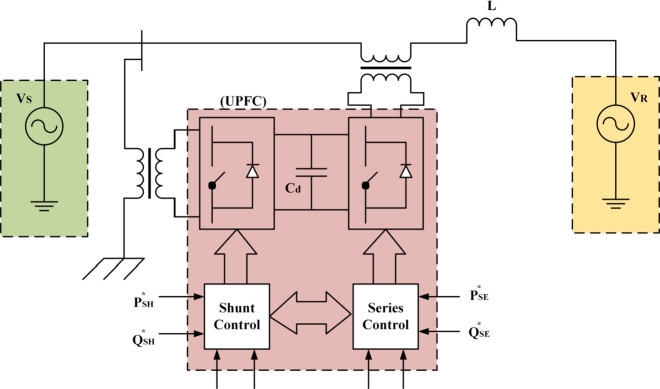
UPQC Microgrid Interconnection for Reactive Power Compensation.

**Figure 2:**
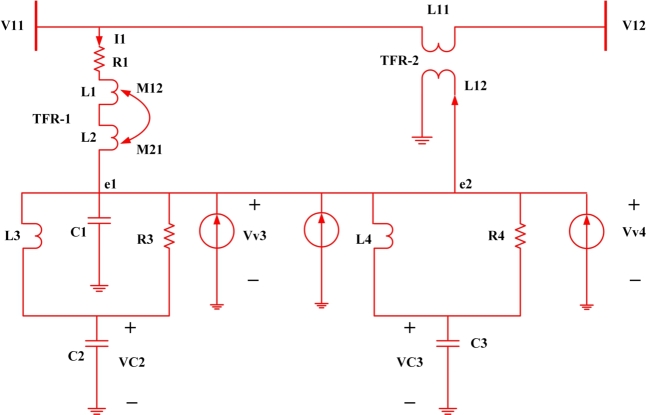
State Space model of UPQC microgrid interconnection.

Therefore in order to develop the model, the system has been analyzed first for the interconnection of the UPQC microgrid for reactive power compensation and is presented in Fig. 1. Here, UPQC shunt and series control has been achieved using the reference value such as series real and reactive power $\left(P_{SE}^{⁎},Q_{SE}^{⁎}\right)$ and shunt real and reactive power $\left(P_{SH}^{⁎},Q_{SH}^{⁎}\right)$, respectively. Again to analyze the stability of the microgrid a state space model of UPQC microgrid interconnection has been developed and presented in Fig. 2.

The dynamic mathematical model for UPQC becomes [33]

(1) $$\left\{ \begin{array}{c} V_{c2}-V_{c3}-V_{1}=u_{f}\\ V_{c3}-V_{c4}-V_{2}=-u_{f} \end{array}\right.$$

Eq.(1), represents the series and shunts compensated output voltage, where $V_{c2}$ and $V_{c3}$ represents the capacitor voltage and $V_{V3}$ and $V_{V4}$ represents the virtual voltage at node-3 and node-4. $u_{f}$ represents the required controllability action as required to compensate the terminal voltage [34]

Again the node-3 and node-4 capacitor voltage is presented at Eq.(2) and Eq. (3) [35]

(2) $$V_{c2}=\int \frac{1}{c_{2}}(\overset{˙}{i_{ds}}-\overset{˙}{i_{dus}})+\int \frac{1}{c_{2}}{\left(\overset{ˆ}{i_{ds}}-\overset{ˆ}{i_{dus}}\right)}^{3}$$

(3) $$V_{c3}=\int \frac{1}{c_{1}}(\overset{˙}{i_{ds1}}-\overset{˙}{i_{vs1}})+L_{3}\frac{di_{L3}}{dt}+R_{3}i_{3}$$

(4) $$V_{1}=R_{1}i_{1}+L\frac{di_{1}}{dt}-M_{12}\frac{di_{12}}{dt}$$

(5) $$V_{c3}=\int \frac{1}{c_{3}}(\overset{˙}{i_{ds3}}-\overset{˙}{i_{dus3}})+\int \frac{1}{c_{3}}{\left(\overset{ˆ}{i_{ds}}-\overset{ˆ}{i_{dus}}\right)}^{3}$$

(6) $$V_{c3}=R_{4}i_{R4}+L_{4}\frac{di_{L4}}{dt}$$

Eq.(4) to Eq.(6), represents the simplified version of node capacitor voltage and that of virtual voltage is presented at Eq.(7)

(7) $$v_{2}=v_{12}$$

Eq.(8) to Eq.(13), represents the state variable equations in terms of VSC1 and VSC2 as a function of capacitance, Inductor and Resistance

(8) $$\overset{˙}{x_{1}}=x_{2}$$

(9) $$\overset{˙}{x_{2}}=int\frac{1}{c_{2}}(\sum (\overset{˙}{i_{ds1}}-\overset{ˆ}{i_{ds1}})+\sum (\overset{˙}{i_{dus1}}-\overset{ˆ}{i_{dus1}}))$$

(10) $$\overset{˙}{x_{3}}=x_{4}$$

(11) $$\overset{˙}{x_{4}}=int\frac{1}{c_{3}}(\sum (\overset{˙}{i_{ds3}}-\overset{ˆ}{i_{ds3}})+\sum (\overset{˙}{i_{dus3}}-\overset{ˆ}{i_{dus3}}))$$

(12) $$\overset{˙}{x_{5}}=x_{6}$$

(13) $$\overset{˙}{x_{6}}=R_{1}i_{1}+L\frac{di_{1}}{dt}-M_{12}\frac{di_{12}}{dt}$$

Now the performance of UPQC can be evaluated in two different stages i.e.capacitor stage, VSC1 stage and VSC2 stage. The capacitor plays a very vital role in the design and control of UPQC. It is controlled by shunt current, capacitance reference voltage and the quadrature component of reference current ($I_{qref.}$).

Again, for the perfect operation of VSC1, every time it has to check the threshold limit of the bus and controller parameter. Therefore,

(14) $$V_{c2}+V_{c3}<(L_{3}\frac{di_{L3}}{dt}+L_{1}\frac{di_{L1}}{dt}+M_{12}\frac{di_{L12}}{dt})$$

Here Eq.(14) represents, the sum of both the capacitor voltage is less than the voltage induced across inductor and parasitic capacitors. Further Eq.(14) can be reduced to Eq.(15)

(15) $$\frac{V_{c2}+V_{c3}}{\left(L_{3}\frac{di_{L3}}{dt}+L_{1}\frac{di_{L1}}{dt}+M_{12}\frac{di_{L12}}{dt}\right)}<1$$

The maximum reactive power that it can inject into the system depends on the controller's action and mathematically presented at Eq.(16)

(16) $$\;\mathrm{mod}\;(q_{1}-q_{vsc1})\leq \delta q_{max}$$

Where $q_{1}$ represents the reactive power demand at bus-1 and $q_{vsc1}$ represents the reactive power injected by the system.

## 3 Solution methodology (controller design)

The backstepping controller can be designed in the following manner. The main objective is to design a suitable controller for the system such that zero error in terms of power compensation can be noticed.

let the set point be $S_{p}^{ref}$ and the corresponding error becomes $\overset{¯}{x_{1}}$ and is presented at Eq.(17).

(17) $$\overset{¯}{x_{1}}=x_{1}-S_{p}^{ref}$$

Now based on Eq.(17), the governing equations are presented at Eq.(18) and Eq.(19)

(18) $$\overset{˙}{\overset{¯}{x_{1}}}=x_{2}-\overset{˙}{S_{p}^{ref}}$$

and

(19) $$\overset{˙}{x_{2}}=L(-e_{1}+v_{f})$$

where

(20) $$L=\frac{1}{\left(L_{s}+R_{s}\right)}$$

and

(21) $$e_{1}=v_{c2}+v_{c3}$$

After introducing the lumped reactance and error at Eq.(20) and Eq.(21), the state space equations are presented from Eq.(22) to Eq.(25)

(22) $$\overset{˙}{\overset{¯}{x_{3}}}=x_{4}-\overset{˙}{S_{p}^{ref}}$$

(23) $$\overset{˙}{x_{4}}=\frac{1}{c_{3}}(\overset{˙}{i_{ds3}-\overset{˙}{\overset{¯}{i_{dus3}}}})$$

(24) $$\overset{˙}{\overset{¯}{x_{5}}}=x_{6}-\overset{˙}{S_{p}^{ref}}$$

(25) $$\overset{˙}{\overset{¯}{x_{6}}}=R_{l}i_{l}+L\frac{di_{1}}{dt}-M\frac{di_{12}}{dt}$$

Again the error available in each path is presented from Eq.(26) to Eq.(31).

(26) $$e_{1}=\overset{¯}{x_{1}}$$

(27) $$e_{2}=x_{2}-\phi_{0}$$

(28) $$e_{3}=\overset{¯}{x_{3}}$$

(29) $$e_{4}=x_{4}-\phi_{1}$$

(30) $$e_{5}=\overset{¯}{x_{5}}$$

(31) $$e_{6}=x_{6}-\phi_{2}$$

Hence the aggregated control action equation becomes,

(32) $$u_{f}=v_{f}+\frac{1}{L}[-k_{6}x_{6}x_{s}-k_{5}x_{5}^{2}x_{4}-k_{4}x_{4}^{4}x_{3}^{2}x_{1}-k_{3}x_{3}^{3}x_{2}^{2}x_{1}-k_{2}x_{2}^{2}x_{1}-k_{1}x_{1}^{2}]$$

Equation (32) represents the final control function which has to be optimized around the set point.

## 4 Experimental setup

In this section, the microgrid architecture has been defined more broadly. To process the proposed controller the microgrid designed offer here has a capacity of 10 kilowatts. The designed microgrid consists of a solar photovoltaic system of 3 Kilowatt and wind system of 4 kilowatt and a fuel cell of 3 Kilowatt. Modeling of photovoltaic cells is required for an efficient design of a PV system. In the proposed architecture MPPT based on P&O algorithm has been used to track the maximum power point. The detailed solar photovoltaic parameters are shown in Table 2.

**Table 2:** Solar PV array Parameter in AC microgrid.

Sr. No.	Parameter	Value
1	Parallel String	8
2	Max. Power	213.15 Wp
3	Open Ckt. Voltage	36.3v
4	Maximum Power Point Voltage	29
5	Temp. Coefficient	-0.3609
6	Short ckt. Current	7.84
7	Current at Maximum Power Point	7.35
8	Temp. Coefficient	0.102
9	Light Generated Current	7.86
10	Diode Ideality factor	0.98
11	Shunt Resistance	313.39 ohm
12	Series Resistance	0.393 ohm

In order to develop a microgrid model of 10 Kilowatts, the IEC TS 62749 code has been adopted. According to it, for inductive based load in an AC microgrid, the power from solar PV should not be more than 32.56% of the total grid capacity. Again according to IEC TR 62510, for 1-Kilowatt of setup up the value for converter, inverter and all other associated grid interconnection parameter values are mentioned.

So from Table 2 it can be concluded that the total solar array capacity is of 3.001 Kwp and that of the terminal voltage is 210 V DC. A boost converter has been modeled to increase the voltage up to 240v so as to make it synchronized with a single phase grid here referred to as a microgrid.

The simulation and experimentation parameters for the designed boost converter is shown in Table 3. Both simulation and design parameter for the boost converter is shown here. The experimental parameters are slightly different from the simulation.

**Table 3:** Simulation & Experimentation parameter for Boost converter.

Sr. No.	Parameter	Rating ((For Simulation)	Rating (For Experimentation)
1	Input Voltage	160v	168v
2	Source Inductance	0.01H	0.015H
3	Source Resistance	1 ohm	1 ohm
4	Source Capacitance	0.002F	0.0022F
5	Load Capacitance	0.002F	0.0022F
6	Load Resistance	24 ohm	24 ohm (Rheostat)
7	Output Voltage	230v	230v

**Figure 3:**
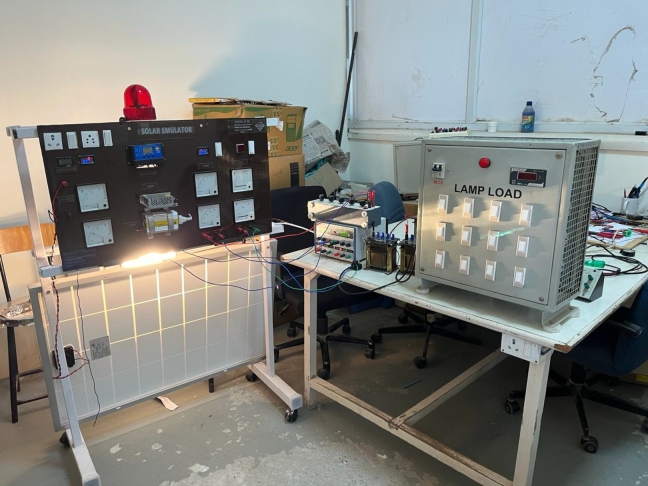
Microgrid Set Up including Solar PV, Wind and Fuel Cell.

The simulated microgrid comprises of 17 Km long transmission line in a radial feeder manner. A switching frequency of 5 KHz has been used in the control logic circuit to produce necessary gating pulses for the central converter. The detailed parameter regarding the same is shown in Table. 4. As shown in Fig. 3 the experimental setup has been prepared in the research laboratory. Different instruments such as Host PC, dSpace, solar emulator, designed prototype and FPGA kit were used for testing the robustness of the proposed controller.

**Table 4:** Converter parameter for AC microgrid.

Sr. No.	Parameter	Rating (Simulation)	Rating(Experimentation)
1	Grid Voltage	220v	230v
2	Length of Tr. Line	17Km	17Km
3	Line Impedance	2+j5.3 Ohm	2+j5 Ohm
4	Turns ration of Transformer	73:2	74:2
5	Switching Frequency	5KHz	4.97KHz.
6	DC-Link Reference Voltage	230V	230V
7	Capacitance of DC-Link Capacitor	2.8mF	3mF
8	Common Mode Inductance	0.1mH	0.11mH
9	Differential Mode Inductance	0.98mH	1.0mH

## 5 Result analysis & discussion

The feasibility study conducted in this section aims to test the effectiveness of UPQC in improving PQ at the Point of Common Coupling (PCC). This section covers a comprehensive analysis of PQ distortion compensation modeling. The main contribution of this section is to propose a method based on backstepping to overcome the limitations of traditional power quality management systems.

Here the effectiveness of the PQ analysis of the microgrid system has been investigated under three different situations.

• Use the backstepping-UPQC method to improve the PQ of a system connected to a microgrid.
• The PQ improvement of the system connected to the Microgrid using the Fuzzy method, with and without UPQC.
• PQ improvement of the system connected to Microgrid through UPQC using the ANFIS method.

### 5.1 Case-1: backstepping

To test the effectiveness of the proposed design method, extensive computer simulations were performed on the uncertainty of the nonlinear system. Different constants for the proposed model as shown in equation (47) were evaluated with Matlab plant fitting model.

**Figure 4:**
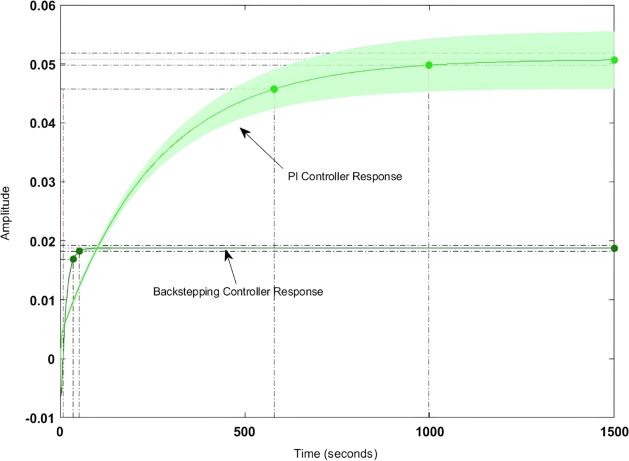
Step response of Backstepping controller.

Fig. 4 shows the step response of the backstepping-PI controller. Here it is found that the peak overshoot has been reduced by 17% and that of the transient stability has been increased by 5.3%. The detailed comparative analysis of the backstepping-PI controller and that of the PI controller is shown in Table 5. Here it can be found that both settling time and rise time have been decreased by 33.2% and 47.4% respectively.

**Table 5:** Comparative analysis between Classical-PI Controller and MRAC-Backstepping controller.

Parameter	Backsteeping-PI Controller	PI-Controller
Settling Time	0.6 sec.	0.82 sec.
Rise Time	0.13 sec.	0.27 sec.
Peak Time	0.18 sec.	0.31 sec.
Peak Overshoot	12.04%	14.3%

**Figure 5:**
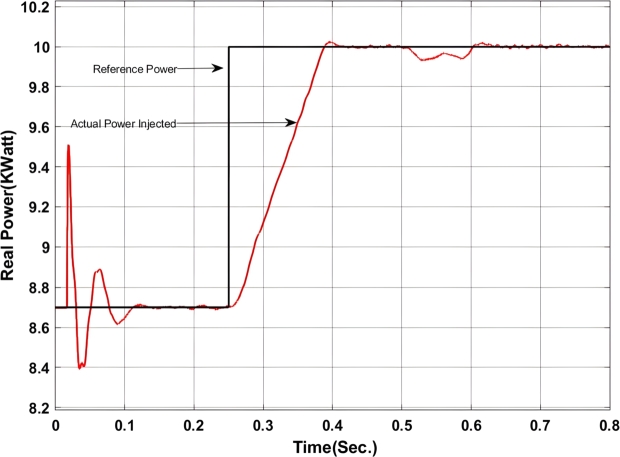
Real power exchange between UPQC and Microgrid with Backstepping PI Controller.

**Figure 6:**
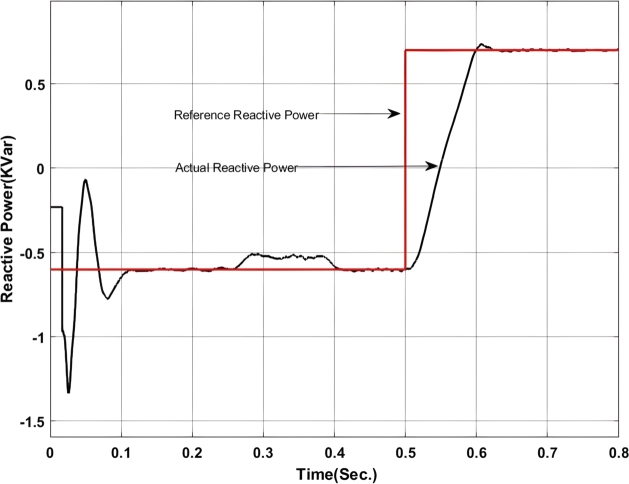
Reactive power exchange between UPQC and Microgrid with Backstepping PI Controller.

With the insertion of backstepping-PI controller the real and reactive power exchanged at the point of common coupling is shown in Figure 5, Figure 6.

Here, from Fig. 5 it can be found that the actual power injected is as per the reference value however it shows some disturbance at the initial stage of operation i.e. up to 0.13 sec. Again from 0.52 sec. to 0.583 sec. it deviated from the pre-defined trajectory this is because of a change in critical load and of the fluctuation in the output of the solar PV system. Similarly, Fig. 6 shows the amount of reactive power exchanged with the Microgrid. It can be found that the system has injected reactive power from 0.5 sec. onward as per the set value and have drawn excessive reactive power from the grid up to 0.5 sec. The reactive power absorbed by the system can be utilized to charge the energy storage device. The maximum amount of reactive power that has been absorbed here is -1.2 KVar.

**Figure 7:**
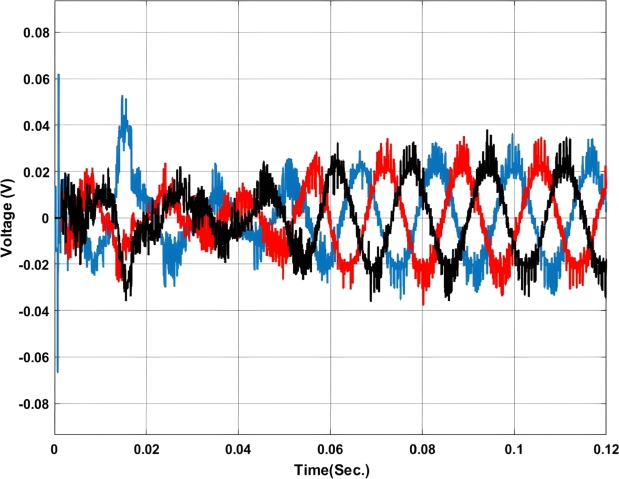
Voltage profile at PCC with Backstepping PI Controller.

**Figure 8:**
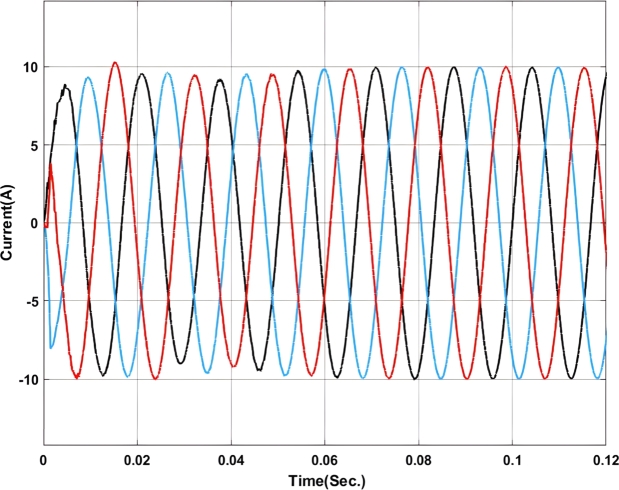
Current profile at PCC with Backstepping PI Controller.

Figure 7, Figure 8 show the voltage and current profile at the PCC. Here it can be found that the voltage waveform is a little bit distorted i.e. up to 0.06 sec. Once the transient period is over the system becomes synchronized and the output voltage is maintained at 230V ac as per the design criteria. In contradiction, the current waveform does not show any distortion.

### 5.2 Case-2:fuzzy-PI controller

In this section, a fuzzy-PI-based current controller for UPQC has been presented. Here 7 membership function based architecture has been adopted for the design of fuzzy rule. Table 6 shows the fuzzy membership and rule table.

**Table 6:** Fuzzy Membership Table.

e	NL	NM	NS	ZE	PS	PM	PL
NL	NL	NL	NM	NM	NS	NS	ZE
NM	NL	NM	NM	NS	NS	ZE	PS
NS	NM	NM	NS	NS	ZE	ZE	PS
ZE	NL	NS	NS	ZE	ZE	PS	PM
PS	NS	PS	ZE	ZE	PS	PM	PM
PM	PS	ZE	ZE	PS	PS	PM	PM
PL	ZE	ZE	PS	PS	PM	PL	PL

In order to decide the controller tuning parameters such as Kp and Ki in a PI Controller. mainly three parameters have been considered viz. regulating bus voltage, controlling the reactive power flow between grid and load and lastly damping power oscillations. Analytical methods like closed loop root-locus technique have been adopted to decide and find the Kp and Ki values. In classical PI controller as the parameters once decided they are fixed, so the Kp and Ki in the case of PI controller is a constant value, therefore, the PI controller remains ineffective in case of transient disturbances.

**Figure 9:**
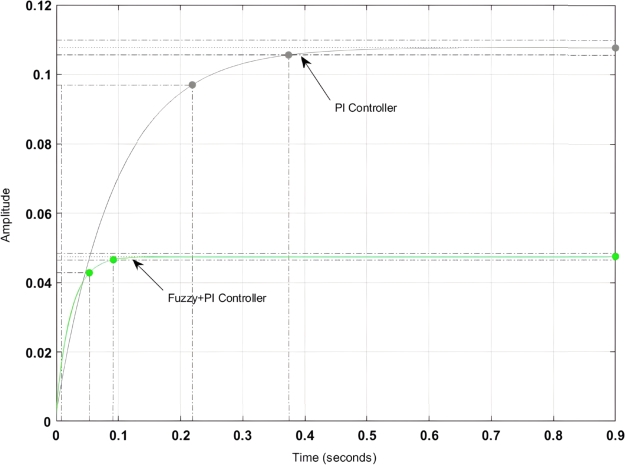
Step response of Fuzzy-PI Controller.

Again, for tuning the Kp and Ki value in a fuzzy logic controller 3-different regions like stable, marginal stable and unstable conditions have been considered. Each region has been allotted with suitable lower boundary and upper boundary for effective optimization of the parameters. The fuzzy-PI based controller has been designed in Matlab controller design. The stability of the controller has been tested with the unit step function and is presented in Fig. 9.

Here it can be found that the steady state stability is better as compared to the classical PI controller however the Fuzzy-PI controller performance is slightly sluggish as compared to the backstepping-PI controller. A detailed comparison among the controllers is presented in Table 7.

**Table 7:** Comparision between Fuzzy-PI and PI-Controller.

Parameter	Fuzzy-PI Controller	PI-Controller
Settling Time	0.77 sec.	0.82 Sec.
Rise Time	0.22 sec.	0.27 sec.
Peak Time	0.28 sec.	0.31 sec.
Peak Overshoot	11.07%	12.04%

Here it can be found that with the fuzzy-PI controller, the settling time has been reduced to 0.77 sec. and that of the peak overshoot has also been reduced to 11.07% as compared to the PI controller.

**Figure 10:**
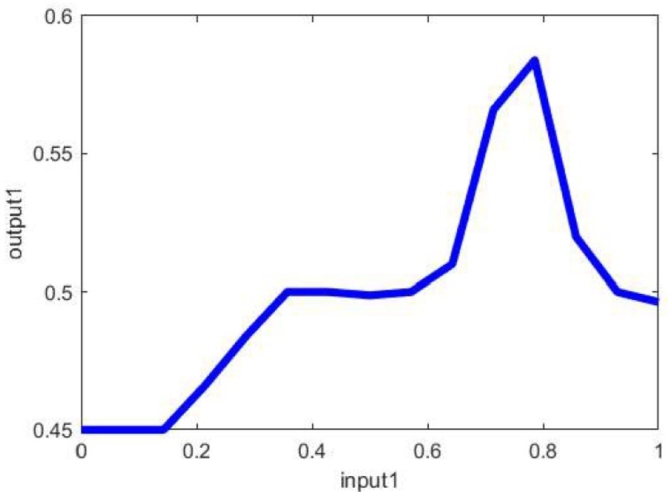
Input to Output mapping of Fuzzy-PI Controller.

Here from Fig. 10, it can be found that the system always tries to tune itself in between 0.55 to 0.583 for an input of 0.6 to 0.83. This is because the Fuzzy-PI controller used here is to stabilize the system under 2.33% of steady-state value which falls under the range of 0.6 to 0.83.

**Figure 11:**
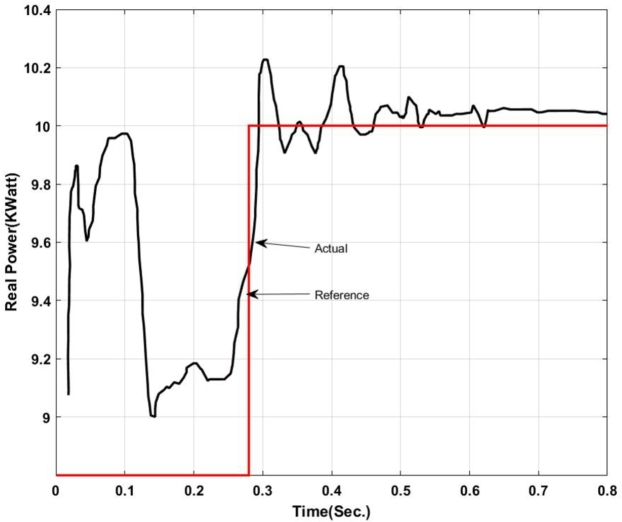
Active Power exchanged with microgrid using Fuzzy-PI Controller.

**Figure 12:**
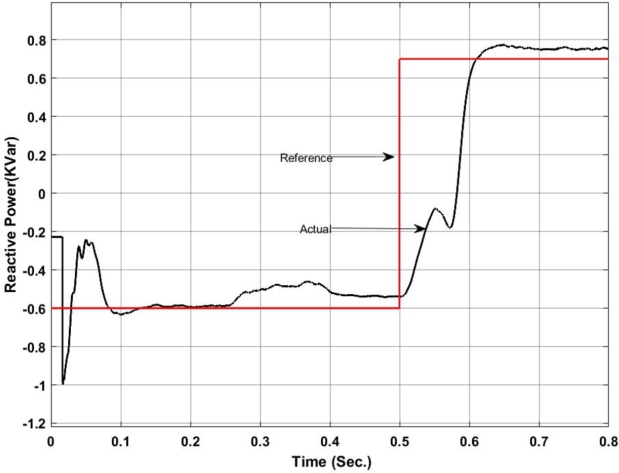
Reactive Power exchanged with microgrid using Fuzzy-PI Controller.

Fig. 11 and Fig. 12 show that active and reactive power is exchanged with the grid using a fuzzy-PI controller. Here it can be noticed that the active power exchanged with the grid fluctuates around the reference value and steady-state behavior in the active power can be noticed after 0.7 sec, with an error rate of 0.142% i.e. the active power inserted is above the reference value and leads to loss of power in the form of cu. loss. Similarly, the reactive power as shown in Fig. 12 also exhibits some deviation in the actual reactive compensation against the trajectory path. Three different notes have been observed in the path. However, the last notch as observed at 0.566 sec is of importance that after this point the reactive power has been injected into the grid as compared to the drawl of reactive power.

**Figure 13:**
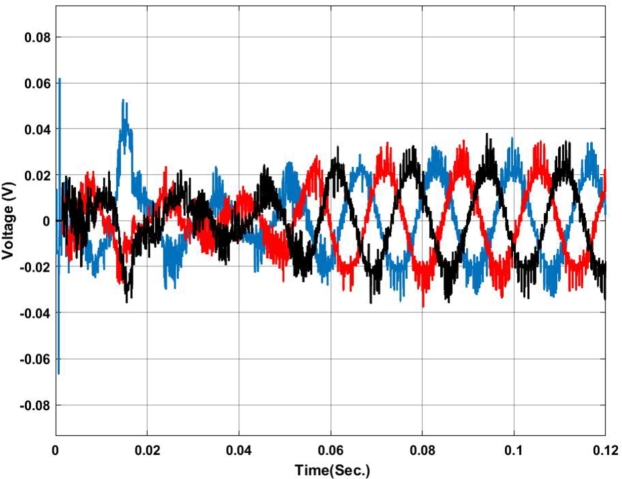
Voltage profile at PCC with Fuzzy-PI controller.

**Figure 14:**
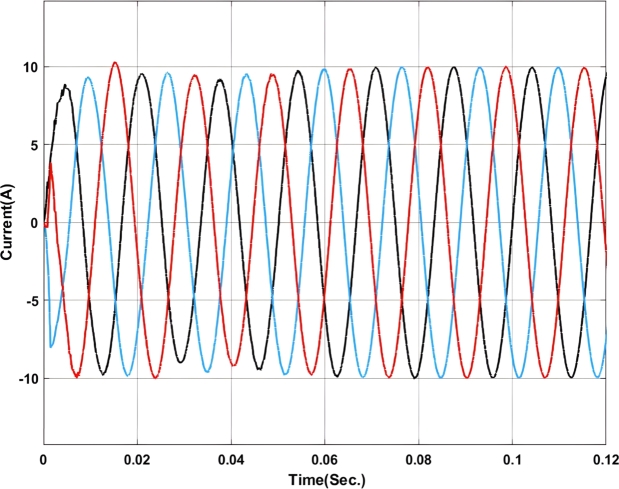
Current profile at PCC with Fuzzy-PI controller.

The voltage and current profiles are presented in Figure 13, Figure 14. The voltage profile exhibits a little bit of oscillation around the set point and the same for the current profile. As compared to backstepping the fuzzy-PI exhibits some distortion in the waveform. After 0.063 sec.the current exhibits almost zero oscillations at the output.

### 5.3 Case-3: ANFIS-PI controller

In this section ANFIS-PI based current controller for UPQC has been presented. ANFIS usually aims to use the prior knowledge of network topology to optimize the space by using the concept of fuzzy neural network logic to improve system performance. The main purpose of the fuzzy logic controller is to check and realize the uncertainty in the system. The functions are chosen in such a way that they correspond to the backpropagation algorithm and make the system tightly adapted to the input and output parameters.

The ANFIS evaluator consists of two parts:-The structure of the evaluator and the training of the evaluator. Design parameters are usually set during the budget stage. Structural parameters usually include input and output membership functions (MF). These membership functions can be triangular, trapezoidal or Gaussian functions. Triangular and Gaussian types of MF are widely used in the literature. In this article, triangular membership functions are also used to define linguistic variables. The backpropagation algorithm is used for training data. Select training parameters through trial and error, such as epoch size, tolerance level, initial step size, and step change rate.

In order to design the ANFIS controller for UPQC, it is required to extract the data from the original model. Therefore, the MATLAB Simulink model was designed and operated at 4-different models such as normal operating conditions, different power flow levels, voltage disturbances and line outages. Again to train the model a CSV file has been created with input parameters to be controlled as bus voltage deviation, Real and Reactive power exchange and line impedance. Similarly, the output parameters of the controller include converter control reference and DC-link reference. So all together 5 parameters were considered for designing the ANFIS controller. Again before proceeding to train ANFIS two data processing techniques such as data normalization and outlinear removal have been carried out for any data redundancy.

Proper tuning of ANFIS parameters (Table 8) is an essential part of enhancing the performance of the controller so in this case MATLAB base Fuzzy Logic Toolbox has been used to tune the parameters. Both the input and output of the controller have been taken into consideration while designing the black box parameters. In this section 52 samples have been examined for designing of optimized ANFIS-PI controller. However, the best 3 samples and their results are shown in Table 9.

**Table 8:** ANFIS-PI optimizer Parameters for Current controller.

Sl. No.	Parameters	Measurement
01	Epoch Size	9
02	Tolerance Level	13.007e-09
03	Initial step size	0.00137
04	Rate of Change of step size	0.0035e-05

**Table 9:** Training of data using Neural Network for direct axis component.

Sl. No.	Parameter	Remarks

Sample-1		
01	Epoch-1	215 Iteration
02	Time in sec	0.02 Sec
03	Performance validation	3.92
04	Gradient(slope)	1.04
05	MU level	1.11
06	Validation Checker	11
07	Plotting Interval	93 epoch
08	Data	Indexing
09	Training time	Levenberg-Marquardt
10	Performance check-1	Mean square error

Sample-2		

01	Epoch-1	213 Iteration
02	Time in sec	0.025 Sec
03	Performance validation	3.97
04	Gradient(slope)	1.03
05	MU level	1.01
06	Validation Checker	17
07	Plotting Interval	97 epoch
08	Data	Indexing
09	Training time	Levenberg-Marquardt
10	Performance check-2	Mean square error

Sample-3		

01	Epoch-2	198 Iteration
02	Time in sec	0.01 Sec
03	Performance validation	3.86
04	Gradient(slope)	1.03
05	MU level	1.01
06	Validation Checker	17
07	Plotting Interval	97 epoch
08	Data	Indexing
09	Training time	Levenberg-Marquardt
10	Performance check-2	Mean square error

**Figure 15:**
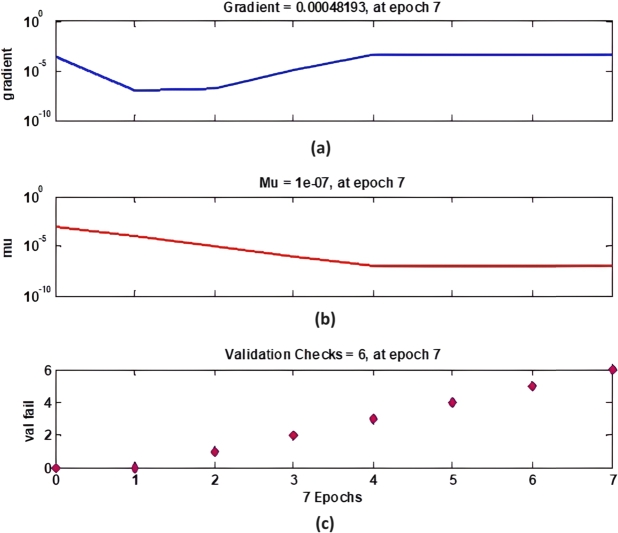
ANFIS Training State for sample-3.

Fig. 15 demonstrates the gradient descent, mu parameter, and validation checks over seven epochs. Sub-Fig. 15. (a) shows the gradient of the training process, which starts at a higher value and decreases, indicating the learning rate and adjustment during the training. Sub-Fig. 15. (b) displays the mu parameter, which gradually decreases, signifying the regularization parameter's adaptation during training. Sub-Fig. 15. (c) presents the validation checks, reflecting the number of validation failures over epochs, which is crucial for understanding the model's performance and overfitting tendency.

**Figure 16:**
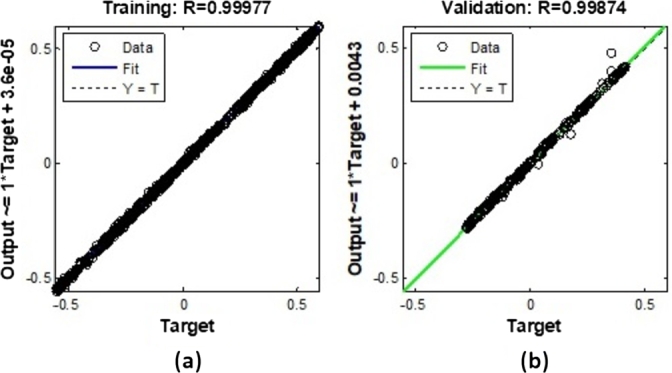
ANFIS NN Regression analysis for sample-3 (a) Training Analysis with R=0.99977 (b) Validation analysis with R=0.99874.

Fig. 16 presents the ANFIS NN regression analysis for sample-3, which is divided into two sub-figures. Sub-Fig. 16. (a) shows the training analysis with a correlation coefficient (R) of 0.99977, while sub-Fig. 16. (b) shows the validation analysis with an R value of 0.99874. These high R values indicate a strong correlation between the predicted outputs and the actual targets, demonstrating the neural network's efficacy in modeling the data accurately during both training and validation phases. The training structure is outlined in Fig. 16, where the execution of the training session is remarkably brief, lasting only 0.01 seconds. This emphasizes the efficiency of the training process. The gradients of the data access components for the three samples are recorded as 0.0048, 0.0051, and 0.0057, with an epoch level of 7 maintained throughout the simulation. This consistency in gradient values signifies a stable learning process. Additionally, the figure includes the data check parameter, which is essential for detecting system redundancy. This parameter is particularly important for the Proportional-Integral (PI) input and output, ensuring that the system operates without unnecessary duplication or errors.

**Figure 17:**
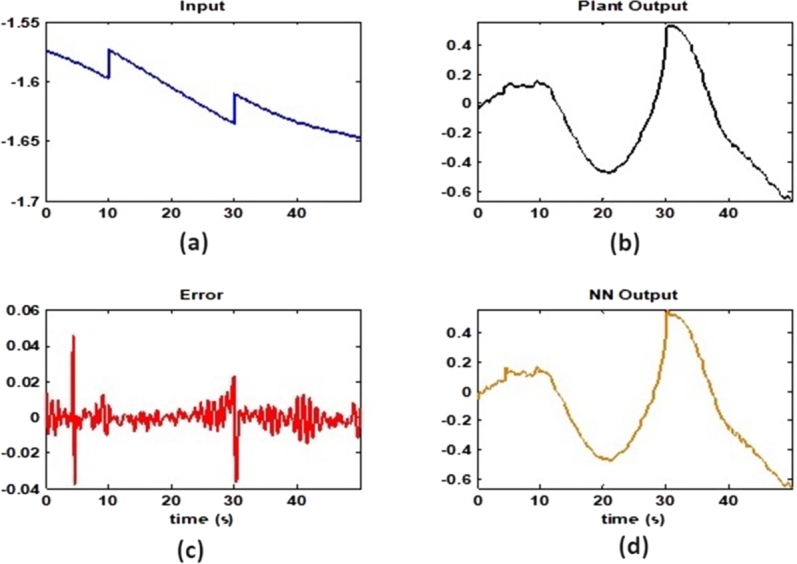
Validation of data using Neural Network for sample-3 (a)Input Signal (b) Output Signal (c) Error rate from Input to Output (d) NN-Output.

Fig. 17 validates the data using a Neural Network for sample-3, comprising four sub-figures that offer a detailed analysis of the input signal, output signal, error rate from input to output, and the neural network's output. Sub-Fig. 17. (a) depicts the input signal, showing a decreasing trend with distinct steps, which represents the changes in the input data over time. This input signal serves as the basis for the neural network to process and generate the corresponding output. Sub-Fig. 17. (b) shows the plant output, which displays a dynamic response with fluctuations, indicating the system's behavior in response to the input signal. This output signal is crucial for understanding how the plant or system reacts to various input conditions. The plant output's accuracy is further analyzed by comparing it with the neural network's output in sub-Fig. 17. (d). The NN output, shown in sub-figure (d), closely follows the plant output, demonstrating the neural network's effectiveness in modeling the system's behavior. Sub-Fig. 17. (c) presents the error rate from input to output, highlighting the discrepancies between the actual plant output and the neural network's predicted output. The error rate analysis is essential for identifying areas where the model can be improved. The figure emphasizes that the typical error range for PI controllers applied to actual equipment is between [0.53-0.827], with brief intervals of 4.3 seconds where deviations occur. The use of the Levenberg-Marquardt algorithm is highlighted, achieving a total throughput of 0.000342 with a slope of 0.00012, indicating its efficiency in optimizing the neural network training process. This analysis reaffirms the consistency and accuracy of the neural network model in handling real-world data and conditions.

**Figure 18:**
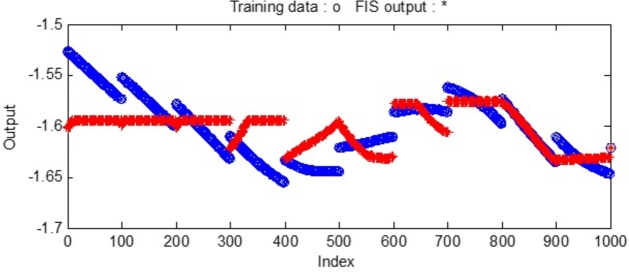
Validation of data using Neural Network for sample-3.

The FIS output (resulting training data) during training of the FIS file using ANFIS is shown in Fig. 18 with 40 no. of Nodes, 27 nonlinear parameters, 1000 training data points and 62 fuzzy rules.

**Figure 19:**
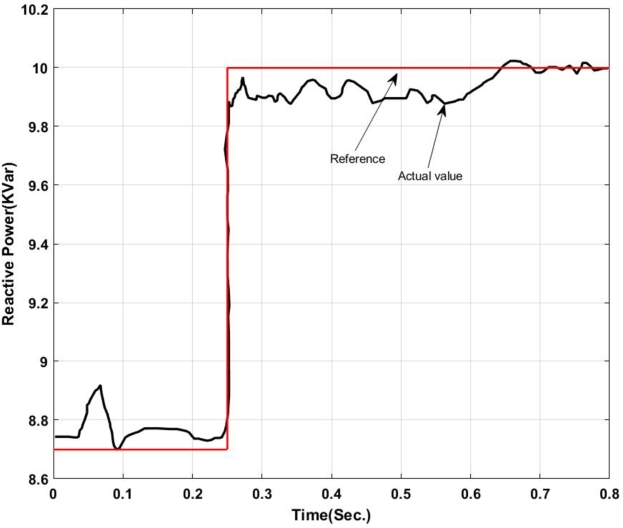
Active Power exchanged with microgrid using ANFIS-PI Controller.

**Figure 20:**
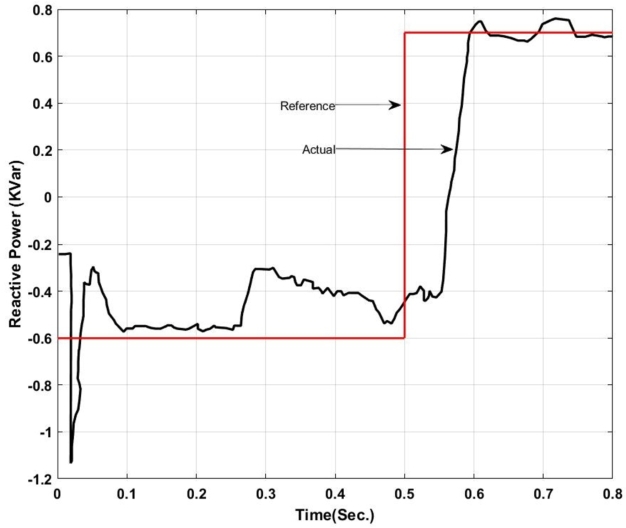
Reactive Power exchanged with microgrid using ANFIS-PI Controller.

A detailed controller check can be initiated by creating a fuzzy inference file and exporting the same file to the workspace for a more detailed analysis of UPQC controller performance. Figure 19, Figure 20 show the active and reactive power exchanged by the UPQC in the microgrid operation. Here the active power exchanged is as per the trajectory from 0.2 sec. to 0.3 sec. After 0.3 sec. the system shows small oscillations in the output power and that of the actual active power exchanged is below the fixed reference value at about 0.7 sec. it again synchronizes to the reference value.

similarly, the reactive power exchange also exhibits oscillations around the reference value. The deviation from the reference value is larger in between 0.3 sec. to 0.48 sec. which can be compared with Fig. 18 i.e. validation of data. Where it can be found that at about 400 and 500 index range a deviation in the predicted and actual occurs in the validation stage. Therefore, the designed controller also exhibits a small deviation in its performance.

**Figure 21:**
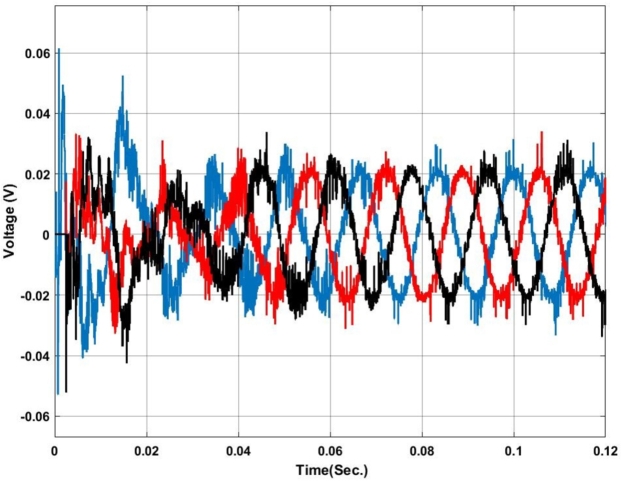
Voltage profile at PCC with ANFIS-PI controller.

**Figure 22:**
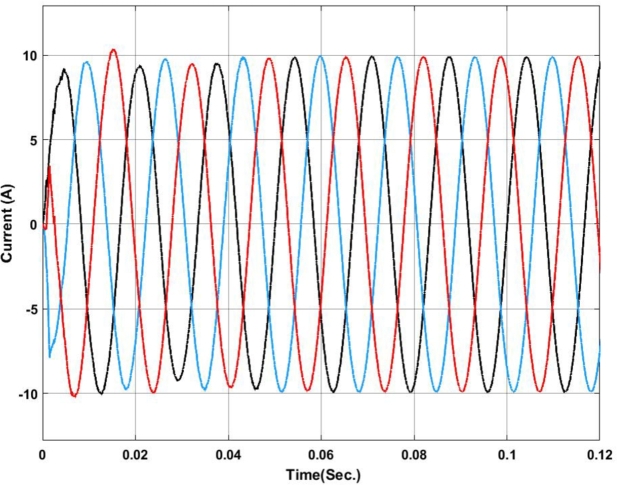
Current profile at PCC with ANFIS-PI controller.

The voltage profile using the ANFIS-PI controller is shown in Fig. 21. It can be found that the system oscillates more around its set point between 0.02 sec. to 0.03 sec. as this is the transient synchronization time for interconnection. Similarly, Fig. 22 shows the current profile of the ANFIS-PI based UPQC for grid interconnection. Here there are no oscillations noticed on the controller performance side.

**Figure 23:**
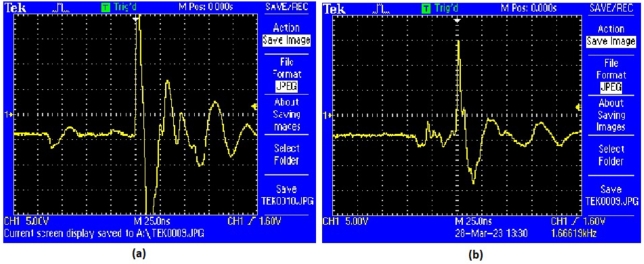
Hardware Controller Performance (a) ANFIS Controller (b) MRAC-Back Stepping Controller.

Fig. 23 provides a comparative analysis of hardware controller performance, showcasing two different controllers: the ANFIS controller and the MRAC-back-stepping controller. Sub-Fig. 23.(a) illustrates the performance of the ANFIS controller, while sub-Fig. 23.(b) depicts the MRAC-back-stepping controller. Both figures display the output signals over time, highlighting key performance metrics such as peak overshoot and settling time, which are critical in assessing controller efficiency and stability.

In sub-Fig. 23. (a), the ANFIS controller's output demonstrates its ability to manage system dynamics effectively. However, sub-Fig. 23. (b) reveals that the MRAC-back-stepping controller achieves a notable improvement in performance by reducing the peak overshoot by 17%. This reduction in peak overshoot is significant as it indicates a better response to disturbances and a more stable control output. The smoother and more controlled response in sub-Fig. 23. (b) suggests that the MRAC-back-stepping controller can handle variations in system input more efficiently.

**Figure 24:**
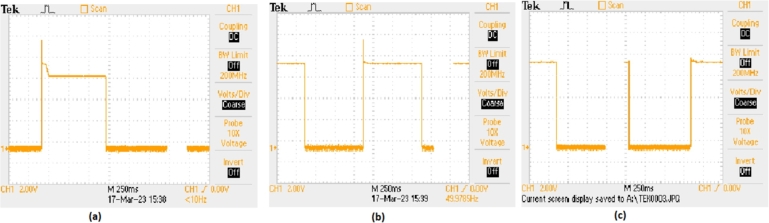
Controller Performance with Respect to Step Input (a) Fuzzy-PI Controller (b) ANFIS-PI Controller (c) MRAC-Back Stepping Controller.

Fig. 24 showcases the performance of different controllers in response to a step input, with each sub-figure representing a different control strategy. Sub-Fig. 24. (a) shows the Fuzzy-PI controller's performance, sub-Fig. 24. (b) presents the ANFIS-PI controller's response, and sub-Fig. 24. (c) depicts the MRAC-back-stepping controller's behavior. These figures highlight the controllers' ability to handle step changes in input, which is crucial for evaluating their effectiveness in real-time applications. In sub-Fig. 24. (a), the Fuzzy-PI controller's performance is shown. The output signal indicates a quick response to the step input, but there are noticeable overshoots and settling time. This suggests that while the Fuzzy-PI controller can respond promptly to changes, it may not be as efficient in minimizing transient behaviors and achieving a stable output quickly. The response characteristics, such as overshoot and settling time, are important parameters for assessing the controller's performance.

Sub-Fig. 24. (b) displays the ANFIS-PI controller's response to the step input. Compared to the Fuzzy-PI controller, the ANFIS-PI controller demonstrates improved performance with reduced overshoot and faster settling time. The smoother transition to the new steady state indicates better handling of the input change, showcasing the advantage of incorporating adaptive neuro-fuzzy inference systems into the PI controller structure. This enhancement is critical for applications requiring precise and stable control. Lastly, sub-Fig. 24. (c) shows the MRAC-back-stepping controller's performance, which exhibits the best response among the three controllers. The MRAC-back-stepping controller shows minimal overshoot and a very short settling time, indicating a highly effective control strategy for step input changes. The superior performance of the MRAC-back-stepping controller highlights its robustness and efficiency in maintaining stability and accuracy, making it a preferred choice for applications demanding high-performance control.

Table-10 shows the Comparative analysis of Controllers interns of stability parameters. As observed, the system is selective and stable under a backstepping controller with 9.88% of overshoot as compared to 11.16% in the case of Fuzzy-PI controller. There is also a marginal difference between the settling time of systems. The settling time for the backstepping controller is 0.63 sec. as compared to 0.71 and 0.80 sec. in the case of Fuzzy and ANFIS PI controllers.

**Table 10:** Comparative analysis of Controllers interns of stability parameters.

Sr. No.	Parameters	Backstopping-PI Controller	Fuzzy-PI Controller	ANFIS-PI Controller
1	Kp	0.41	0.46	0.52
2	Ki	0.22	0.25	0.28
3	Rise Time (Sec.)	0.06	0.07	0.08
4	Peak Time (Sec.)	0.11	0.12	0.14
5	Delay Time (Sec.)	0.09	0.10	0.11
6	Settling Time (Sec.)	0.63	0.71	0.80
7	Maximum Overshoot (%)	9.88	11.16	12.62
8	Under Shoot(%)	23.01	26.00	29.38
9	Stability	Stable	Marginally Stable	Marginally Stable

**Figure 25:**
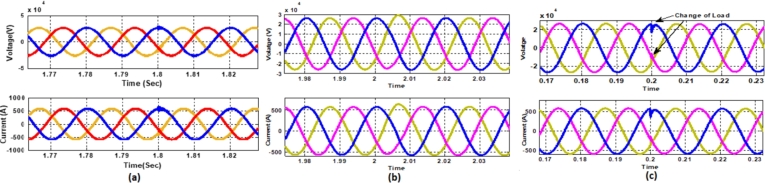
Comparative analysis of Fault Detection among controllers for Voltage and Current waveform at PCC under L-G Fault (a) MRAC-Backstepping PI-Controller (b) ANFIS-PI Controller (c) Fuzzy-PI Controller.

Fig. 25 presents a comparative analysis of fault detection among various controllers for voltage and current waveforms at the point of common coupling (PCC) under a Line-to-Ground (L-G) fault condition. Sub-Fig. 25. (a) shows the performance of the MRAC-Backstepping PI-Controller. The voltage and current waveforms indicate a stable response with clear fault detection. This controller effectively manages the system dynamics, with the fault detected in 0.04 seconds. The rapid detection and response highlight the efficiency and robustness of the MRAC-Backstepping PI-Controller in maintaining system stability and quickly addressing faults. Sub-Fig. 15. (b) depicts the ANFIS-PI Controller's response to the same fault condition. While the ANFIS-PI Controller also demonstrates effective fault detection, it takes slightly longer, at 0.044 seconds, to identify the fault. The voltage and current waveforms show a stable but slightly delayed response compared to the MRAC-Backstepping PI-Controller. This indicates that the ANFIS-PI Controller, while effective, may not be as fast in detecting faults as the MRAC-Backstepping PI-Controller. Sub-Fig. 25. (c) presents the Fuzzy-PI Controller's performance. The fault detection time for this controller is 0.054 seconds, the longest among the three controllers analyzed. The voltage and current waveforms show a more delayed response, indicating that the Fuzzy-PI Controller is less efficient in detecting faults quickly. This comparative analysis underscores the superiority of the MRAC-Backstepping PI-Controller in terms of rapid fault detection and response, making it the most effective controller for maintaining system stability under fault conditions.

Table-11 represents a comparative analysis of fault detection time between backstepping, fuzzy logic, and ANFIS controllers. While the performance and effectiveness of each controller depend on specific applications, system dynamics, tuning parameters, and implementation details, certain considerations can be made. Backstepping controllers, designed for nonlinear systems, use residual-based techniques for fault detection and can achieve fast detection times due to their ability to handle nonlinearities. Fuzzy logic controllers rely on linguistic variables and rule-based inference, but their fault detection time may not always match that of backstepping controllers. ANFIS controllers, combining neural networks and fuzzy logic, adapt through a learning algorithm but may not consistently achieve the same fault detection speed as backstepping controllers. To enhance the backstepping controller's fault detection time, factors such as accurate system Modeling, robust observer design, proper residual generation, threshold setting, tuning parameter optimization, and thorough validation/testing should be considered, with the understanding that the specific system and application requirements play a significant role in determining overall controller performance.

**Table 11:** Comparative analysis of controller performance in terms of fault detection.

Sr. No.	Controller Type	Distance from PCC (km)	Fault Detection Time		
			L-Fault	LL-Fault	LLL-Fault
1	**Table:** Backstepping-PI Controller	5	0.040	0.080	0.110
		10	0.043	0.086	0.119
		15	0.049	0.098	0.134

2	**Table:** ANFIS-PI Controller	5	0.044	0.089	0.122
		10	0.052	0.105	0.144
		15	0.061	0.122	0.167

3	**Table:** Fuzzy-PI Controller	5	0.054	0.108	0.148
		10	0.067	0.133	0.183
		15	0.079	0.159	0.218

As noticed the proposed MRAC-Back-stepping controller is a mathematically driven method, which has an adaptable nature as compared to other fuzzy and ANFIS-PI controllers. The fuzzy and ANFIS-PI utilize the historical data to compensate for the system uncertainties. Therefore, the adaptability and robustness of the model have been increased in the case of MARC driven back-stepping controller.

## 6 Conclusion

Maintaining the proper voltage in the power system is the most important problem that must be solved for any type of power system problem. System failures and drops in terminal voltage are unacceptable from the stability point of view of the microgrid. The system should be designed to work within a certain period, or else after 50 cycles permanent interruption may occur. Advances in power electronic control have made it possible to manage these conditions without interrupting system operation. To avoid further voltage collapse UPQC is connected with some protective measures to the weakest bus as identified by load flow solutions

In this paper backstepping, fuzzy logic, and ANFIS based PI controller for the inner current control loop have been demonstrated. The Matlab-based result thus obtained for three different controllers in a microgrid has been described separately. As seen under the result section, the stability in the case of MARC-Back-stepping controller is highly stable with a maximum overshoot of 9.88% and settling time of 0.63 sec. which is the lowest among the others. Similarly, the fault detection time for L-G fault in MRAC-back-stepping controller (5 km) is 0.04 sec., LL-G fault is 0.08 and 0.11 sec. in the case of LLL-G fault. This shows that, how dynamically the proposed controller is working under different conditions of fault.

## Funding Statement

No funding was supported for this research work.

## CRediT authorship contribution statement

**Sandip Kumar Das:** Writing – original draft, Validation, Software, Methodology, Formal analysis, Data curation, Conceptualization. **Sarat Chandra Swain:** Writing – review & editing, Supervision, Software, Resources, Methodology, Investigation, Formal analysis, Data curation, Conceptualization. **Ritesh Dash:** Writing – review & editing, Writing – original draft, Visualization, Software, Resources, Methodology, Investigation, Formal analysis. **Jyotheeswara Reddy K:** Writing – review & editing, Writing – original draft, Visualization, Validation, Software, Methodology, Investigation, Formal analysis, Data curation. **Dhanamjayalu C:** Writing – review & editing, Validation, Supervision, Software, Resources, Project administration, Investigation, Conceptualization. **Ravikumar Chinthaginjala:** Writing – review & editing, Writing – original draft, Visualization, Validation, Data curation. **Ramakanta Jena:** Writing – review & editing, Writing – original draft, Visualization, Validation, Data curation. **Hossam Kotb:** Writing – review & editing, Writing – original draft, Visualization, Validation, Resources, Formal analysis, Data curation. **Ali ELrashidi:** Writing – review & editing, Writing – original draft, Visualization, Methodology, Investigation, Formal analysis.

## Declaration of Competing Interest

The authors declare that they have no known competing financial interests or personal relationships that could have appeared to influence the work reported in this paper.

## Data Availability

No data is available in this article.
